# Outcomes of Sinus Laser Therapy in Sacrococcygeal Pilonidal Sinus Disease: A Single-Center Experience

**DOI:** 10.7759/cureus.29388

**Published:** 2022-09-21

**Authors:** Ahmad M Zubaidi, Mohammed N Alali, Sulaiman A AlShammari, Abdulrahman H Zikry, Mohammed Habib, Abdulaziz S AlSalem, Mohamed H Sirelkhatim, Reem Alharbi

**Affiliations:** 1 Colorectal Research, Department of Surgery, College of Medicine, King Khalid University Hospital, King Saud University, Riyadh, SAU; 2 Department of Surgery, Prince Mohammed Bin Abdulaziz Hospital, Second Health Cluster in Central Region, Ministry of Health, Riyadh, SAU; 3 Department of Clinical Surgery, College of Medicine, Princess Nourah Bint Abdulrahman University, King Abdullah Bin Abdulaziz University Hospital, Riyadh, SAU

**Keywords:** silac, pilonidotomy, silat, laser therapy, spnd, sacrococcygeal pilonidal disease

## Abstract

Sacrococcygeal pilonidal sinus disease (SPND) is an acquired chronic disease with no precise etiopathogenesis. The morbidity associated with the disease necessitates the implementation of new techniques, such as sinus laser therapy (SiLaT), to improve disease management. However, surgical techniques as of now are preferred as the mainstay mode of treatment.

A retrospective study was conducted to evaluate and report the healing outcome of the application of SiLaT on patients with SPND at a tertiary center. All patients who underwent SiLaT for primary or recurrent pilonidal sinus from February 2012 to December 2019 were included in the study and followed up for at least six months. Forty-one participants (37 males (90.2%) and four females (9.8%)) were included. Of the participants, 58.5% presented with chief complaints of painful swelling with mucopurulent discharge. Most of the participants were students (43.9%). SiLaT was the primary intervention for 82.9% of the participants. The mean duration of hospital stays, resumption of regular activity, and complete wound healing by secondary intention were 30±21.5 hours, 18.4±14.3 days, and 6.5±6.6 weeks, respectively. Around 95.1% of wounds healed without complications. The overall recurrence rate was 24.4%, while the recurrence rate with SiLaT being the primary intervention was 11.8%. Only three (7.32%) patients experienced wound infections as postoperative complications. The visual analog scale (VAS) score decreased postoperatively in the first and second weeks to 3.9±3.2 and 1.9±1.9, respectively, and 78.1% of the total patients showed satisfaction post-surgical interventions. The current study showed that the SiLaT technique is a feasible technology with promising results to evolve. Further studies are encouraged.

## Introduction

Sacrococcygeal pilonidal sinus disease (SPND) is an acquired disease with no precise etiopathogenesis. It is mainly related to the recurrent erosion of hair follicles, resulting in a tract or cavity at the natal cleft (the sacrococcygeal region) [1-3]. The most common complaints are pain, discharge, bleeding, and itchiness around the sacrococcygeal region. These can be acute or chronic issues with disease severity ranging from small, asymptomatic openings to multiple tracts and fistulization away from the midline [3-5]. Obesity, hirsutism, deep gluteal cleft, sitting for several hours per day, and family history are the most reported risk factors [3]. It is noted that hair removal using laser, depilatory creams, waxing, and shaving was associated with a reduction in the recurrence rate of SPND [2,3,5]. For unknown reasons, SPND incidence has been increasing in the past decades. It is estimated to be 26 cases per 100,000 inhabitants, reaching up to 46 cases/100,000, with a higher prevalence among males and individuals aged between 15 and 30 years [1,5].

A gold standard definitive treatment for SPND remains controversial despite the variety of available surgical options [1]. Since there is a significant negative impact of SPND on the quality of life of affected subjects even after surgical management, new management techniques have become need of the hour, such as sinus laser therapy (SiLaT) or sinus laser-assisted closure (SiLaC). It has been evolving in the past few years with promising outcomes and reasonable patient satisfaction [6,7]. It is essential to know that SiLaT works by introducing a radial fiber connected to a diode laser into the sinus to eradicate its cavity after extracting the hair, and the sinus is curetted [5].

A limited number of SiLaT studies exist in the literature, with no single study reported from Saudi Arabia or our Gulf states. Therefore, the aim of the current study is to report the first local Saudi experience using such a minimally invasive technique in the management of SPND by assessing the outcome on the Saudi population and comparing it with worldwide reports.

## Materials and methods

Study setting, design, and procedure

A single-center, retrospective, cross-sectional study was carried out from February 2012 to December 2019 using our colorectal center’s database and our electronic filing system (Electronic System for Integrated Health Information (e-SIHI)) for evaluating the impact of SiLaT on outcomes in SPND at King Khalid University Hospital, a tertiary care hospital in Saudi Arabia. Approval from the institutional ethical committee was obtained for conducting this study (reference number 20/0078).

The study participants were patients who came to our colorectal surgery outpatient clinic seeking surgical care for sacrococcygeal pilonidal disease and were treated electively with SiLaT. The total number of reviewed patients was 56 (15 were excluded as they were anal fistula and hemorrhoid cases). All cases were examined by a single surgeon specializing in colorectal surgery and perianal diseases (AZ). The inclusion criteria were all patients who underwent SiLaT for pilonidal sinus, even redo patients (not the primary intervention) with regular postoperative follow-ups until discharge from the clinic, or those whose charts did contain the needed data for the study but did follow the instructions. Meanwhile, the exclusion criteria were patients who missed the regular postoperative follow-ups or whose charts did not contain the needed data for the study, did not follow instructions, or refused to participate.

Patient demographics and clinical characteristics were collected for all eligible patients, including patient’s presentation, history of abscess drainage, duration of complaint before SiLaT, type of intervention (primary versus secondary), duration of hospital stays, resumption of regular daily activities, duration of complete wound healing by secondary intention, preoperative and postoperative pain (use of analgesics and the visual analog scale (VAS) for pain scores) [8], smoking, complications, recurrence (presence of any of the following after complete wound healing: redness, swelling, pain, new opening, or discharge), and any intervention after recurrence. The severity of complications was categorized according to the Clavien-Dindo classification [9]. All patients were interviewed over the phone after obtaining verbal consent to confirm the recurrence status and assess their satisfaction by a single trained data collector (AHZ) through the use of the Likert scale/score assessment tool, which was utilized in multiple published papers [7,10]. The Strengthening The Reporting Of Cohort Studies in Surgery (STROCSS) guidelines have been followed in this project [11]. We did not follow any specific criteria to include the patients, and SiLaT was offered as one of the management choices for all patients with noninfected SPND. All cases of SiLaT were performed with a single session of laser therapy, under general anesthesia, in the jackknife position after shaving and sterilizing the back and receiving a single IV dose of cefazolin. Laser therapy sessions were performed through the use of a Leonardo Dual 45 Laser device (biolitec biomedical technology, Jena, Germany). Laser emissions were delivered through a 360° radial fiber connected to a diode laser machine that was set at a wavelength of 1420 nm and energy of 10 Watts. It was used at 10 J/cm^2^ fluences with a 5 ms pulse duration.

Statistical analysis

Descriptive analyses were utilized for reporting frequencies and proportions to describe the characteristics of the study population for categorical variables such as gender and surgical outcomes. Mean was used for continuous variables, including the age of the patients and the length of stay (days) in the hospital. All analyses were performed using IBM Statistical Package for the Social Sciences (SPSS) Statistics version 25.0 (IBM Corp., Armonk, NY, USA).

## Results

The study included 41 participants, most of whom had no chronic illness or positive family history of SPND. Table 1 demonstrates the demographic characteristics of the patients. Most participants were males (90%), with a mean age of 22.8±5.9 years. The body mass index (BMI) of the study population was 27±5.2 kg/m². Most patients presented with discharge and pain (58.5%) with a duration of complaint before surgery of 15.3±24.6 months. Most patients (61%) presented with a history of surgical drainage. The most dominant participant occupation was that of being a student, followed by an office worker, while most were nonsmokers (51.6%). Thirty-seven (90%) patients had two to three SPND openings. An average energy of 800±200 J was delivered as a treatment to the patients.

**Table 1:** Demographic characteristics of the patients. SD: standard deviation; BMI: body mass index

Variables		Number (%)
Age	Mean±SD	22.8±5.9
Gender	Male	37 (90.2)
	Female	4 (9.8)
BMI	Mean±SD	27±5.2
Presentation	Discharge and/or pain	24 (58.5)
	Discharge	13 (31.7)
	Pain	2 (4.9)
	Swelling	2 (4.9)
Duration of complaint before surgery (months)	Mean±SD	15.3±24.6
Presence of abscess surgical drainage history	Yes	25 (61)
	No	16 (39)
Occupation	Student	18 (43.9)
	Lawyer	1 (2.4)
	Doctor	1 (2.4)
	Banker	1 (2.4)
	Office worker	7 (17.1)
	Field worker	1 (2.4)
	Engineer	2 (4.9)
	Soldier	2 (4.9)
	Businessman/businesswoman	2 (4.9)
	Unemployed	2 (4.9)
	Accountant	1 (2.4)
	Technician	1 (2.4)
	Housewife	1 (2.4)
	Pilot	1 (2.4)
Smoking	Yes	18 (43.9)
	No	23 (56.1)

All participants were treated under general anesthesia. The mean duration of follow-up after SiLaT was 35.3 months. Table 2 presents the characteristics of the intervention and the presence of complications. SiLaT was the primary intervention for most participants (82.9%) in comparison with the secondary intervention (17.1%). The mean duration of hospital stay was 30±21.5 hours, while the mean duration until the resumption of regular activity was 18.4±14.3 days. The mean duration of complete wound healing by secondary intention was 6.5±6.6 weeks. Around 95.1% of wounds healed without complication. The overall recurrence rate was 24.4%. Furthermore, the recurrence rate with SiLaT being the primary intervention is 11.8%, while it is 42.9% with SiLaT being the secondary intervention. Only three (7.32%) patients in the current study experienced postoperative complications, which were wound infections and were graded on Clavien-Dindo classification as grade II (100%).

**Table 2:** Characteristics of the intervention and presence of complications. SiLaT: sinus laser therapy; SD: standard deviation

Variables		Number (%)
Type of laser intervention	Primary (SiLaT)	34 (82.9)
	Secondary	7 (17.1)
Duration of hospital stay (hours)	Mean±SD	30±21.5
Resumption of normal activities (days)	Mean±SD	18.4±14.3
Duration of complete wound healing by secondary intention (days)	Mean±SD	6.5±6.6
Wound complications	Yes	3 (7.3)
	No	39 (92.7)
Overall recurrence (6-95 months)	Yes	10 (24.4)
	No	31 (75.6)
Recurrence within nonrecurrent cases (laser as the primary intervention)	Yes	4 (11.8)
	No	30 (88.2)
Recurrence within the recurrent cases (laser as the secondary intervention)	Yes	3 (42.9)
	No	4 (57.1)
Type of intervention after recurrence	Skin flab	1 (10)
	Redo laser	1 (10)
	None	8 (80)

**Figure 1:**
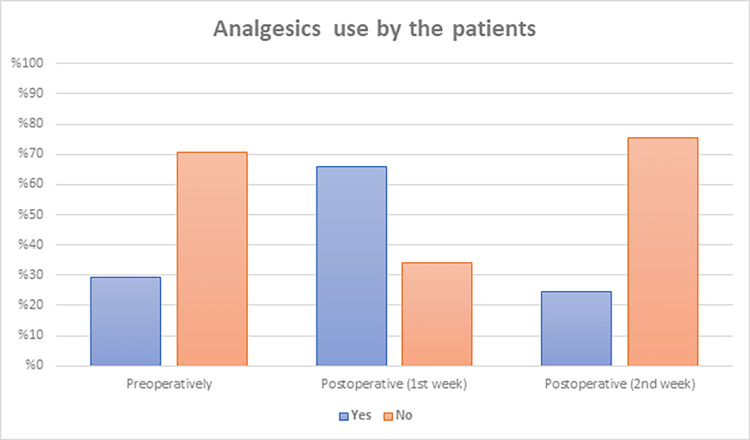
Analgesic use by the patients.

Figure 1 presents the analgesics used by patients as around one-fourth of the patients did not use analgesics preoperatively (29 patients) and two weeks postoperatively (31 patients). On the contrary, most patients used analgesics in the first week postoperatively (27 (65.9%) patients). Table 3 demonstrates the patients’ pain perception and satisfaction before and after the intervention. The mean preoperative visual analog scale (VAS) score was 4.4±3.8, and the score decreased postoperatively in the first and second weeks to 3.9±3.2 and 1.9±1.9, respectively. Following SiLaT, using the Likert scale/score assessment tool, most participants were very satisfied (68.3%), while a few were very dissatisfied (12.2%), since most of them experienced complications.

**Table 3:** Patients’ pain perception and satisfaction before and after treatment. VAS: visual analog scale; SD: standard deviation

Variables		Number (%)
VAS score		
Preoperative	Mean±SD	4.39±3.83
Postoperative (first week)	Mean±SD	3.93±3.16
Postoperative (second week)	Mean±SD	1.93±1.93
Patient satisfaction index	Mean±SD	4.22±1.4
	Very satisfied	28 (68.3)
	Satisfied	4 (9.8)
	Neutral	4 (9.8)
	Dissatisfied	0 (0)
	Very dissatisfied	5 (12.2)

## Discussion

The effect of the treatment of SPND on the patient’s psychology and quality of life imposes a significant burden on the physician to implement new methods to improve the disease’s management. However, treating SPND using surgical techniques is still the standard of care. SiLaT is one of the promising techniques of the growing trend toward minimally invasive treatment of SPND, but it needs to be further investigated and validated, which is the main aim of the current study. The demographic characteristics of the patients were similar to those reported in the literature [1,2,4-6,10,12,13].

The use of SiLaT is straightforward, with a short cutting time (10-15 minutes) and hospital stay. The hospital stay is nonrepresentative, as most of the patients were admitted four to six hours prior to surgery and discharged when it was appropriate, four to six hours after surgery, unless the patient was not from the town or their conditions necessitated their stay in the hospital for another day. The procedure can be performed for all types of SPND under locoregional anesthesia or local sedation [6]. However, since the jackknife position in perianal cases is our preference, it was performed under general anesthesia.

The mean duration for the resumption of regular activities following SiLaT in the current study was higher (18.4 days) than that reported in multiple studies in the literature [5,6,14]. Moreover, the mean wound healing rate by secondary intention was low after SiLaT (6.5 days), which is better than the reported mean wound healing rate [5,6,14]. The current study had a low percentage of postoperative complications (7.3%). It only involved wound infection compared to those reported from surgical intervention, which involved wound infection, dehiscence, and seroma. All the complications seen individually in three cases were treated conservatively and graded on Clavien-Dindo classification as grade II [15]. At the same time, SiLaT studies by Pappas and Christodoulou [14] and Abdelnaby et al. [5] reported similar rates but different types of postoperative complications (wound infection only versus wound infection and hematoma, respectively) in comparison with the current study. On the contrary, Dessily et al. [6] reported a higher rate and different types of postoperative complications (wound infection, fibrin membrane, and hematoma) in comparison with the current study.

The worldwide recurrence rate of surgical management of SPND is varied and reported in some studies to be as lower as 0.2%, with the best result achieved using modified Limberg flap [2,3]. Albabtain et al. [15] and Almajid et al. [16] reported a recurrence rate of 22.8% and 7.2%, respectively, for patients treated by surgical excision of SPND in the Kingdom of Saudi Arabia, while Abdelnaby et al. [5] reported zero recurrence rate of sinus lay open technique in Egypt [2]. With a relatively good follow-up duration (6-95 months), the overall recurrence rate of SiLaT in the current study is 24.4%, which is considerably high in comparison with different types of laser therapy techniques in the literature (0%-20%) [2,4,6,12,14,15]. This rate can be related to the nature of the study population, including all types of SPND, even recurrent cases. Moreover, with such a blind procedure, some trapped hair or lateral tracts can be missed. However, if cases of SiLaT being the secondary intervention were excluded, the recurrence rate of the current study is 11.8%, which is comparable to multiple studies in the literature. However, our success rate in treating recurrent cases (57.1%) is lower than the literature (75%-100%) [6,13,14]. Some of those who had recurrence and agreed to undergo another surgical intervention in our institution (20%) were treated with either redo laser therapy (10%) or skin flap (10%) with no reported further recurrence.

While pain killer usage and visual analog scale are not the optimal methods to assess pain, they showed a slightly lower percentage of using pain medications with good pain scores in the perioperative period in comparison with other studies. Furthermore, patient satisfaction was comparable to other studies as most patients were very satisfied with the outcomes [5,6]. The cornerstone in developing SPND is hair at the natal cleft area. Therefore, since there is good evidence of laser depilation, all patients were instructed to take laser depilation sessions postoperatively, and they were found to have 100% compliance [17-19].

Strengths and limitations

This cross-sectional study adds to the literature about outcomes and patient satisfaction with SiLaT for SPND. So far, to the best of our knowledge, no single study has been reported from Saudi Arabia or the nearby Gulf states. There are multiple limitations to this study, including being a retrospective study (future prospective studies are needed) and a single-institution study (a sizeable multicenter study is needed), with a small sample size (a larger sample size is needed in future projects).

## Conclusions

A picture of using the SiLaT technique for SPND in the Kingdom of Saudi Arabia is reported. It showed that the SiLaT technique is a feasible technology that might help in decreasing the negative impact of SPND on the quality of life of affected subjects and has generally good outcomes. Further future studies are encouraged to have more evidence and better assessments of such emerging procedures that might replace surgical techniques.
